# Custo-Efetividade do Emprego do Escore de Cálcio Coronariano na Orientação para a Decisão Terapêutica na Prevenção Primária, na População Brasileira

**DOI:** 10.36660/abc.20210347

**Published:** 2022-06-06

**Authors:** Raul Serra Valério, Giuliano Generoso, Juliano Lara Fernandes, Khurram Nasir, Jonathan C. Hong, Marcio Sommer Bittencourt

**Affiliations:** 1 Diagnósticos da América SA São Paulo SP Brasil Diagnósticos da América SA, São Paulo, SP – Brasil; 2 Hospital Sírio-Libanês Centro de Cardiologia São Paulo SP Brasil Hospital Sírio-Libanês - Centro de Cardiologia, São Paulo, SP – Brasil; 3 Radiologia Clínica de Campinas Campinas SP Brasil Radiologia Clínica de Campinas, Campinas, SP – Brasil; 4 Instituto de Ensino e Pesquisa Jose Michel Kalaf Campinas SP Brasil Instituto de Ensino e Pesquisa Jose Michel Kalaf, Campinas, SP – Brasil; 5 Houston Methodist Debakey Heart & Vascular Center Center for Outcomes Research Houston Texas EUA Houston Methodist Debakey Heart & Vascular Center - Center for Outcomes Research, Houston, Texas – EUA; 6 Texas Heart Institute Division of Cardiovascular Surgery Texas EUA Texas Heart Institute - Division of Cardiovascular Surgery, Texas – EUA; 7 Universidade de São Paulo Hospital Universitário de Sao Paulo São Paulo SP Brasil Universidade de São Paulo - Hospital Universitário de Sao Paulo, São Paulo, SP – Brasil

**Keywords:** Análise Custo-Benefício, Prevenção Primária, Técnicas de Imagem Cardíaca, Cálcio Coronariano

## Abstract

**Fundamento:**

O emprego do escore de cálcio no auxílio da estratificação de risco cardiovascular pode ser ferramenta com melhor custo-efetividade em comparação à estratégia convencional.

**Objetivos:**

Avaliação da custo-efetividade do emprego do escore de cálcio na orientação terapêutica para a prevenção primária cardiovascular.

**Métodos:**

Modelo de microssimulação para avaliar as consequências clínicas e econômicas da doença cardiovascular aterosclerótica, comparando-se a estratégia de prevenção pelo uso do escore de cálcio e a estratégia convencional.

**Resultados:**

Resultados obtidos demonstram melhor custo-efetividade da estratégia terapêutica guiada pelo escore de cálcio, por meio da redução do custo incremental, e aumento nos anos de vida ajustados por qualidade (QALY), que corresponde, em número, ao benefício incorporado à qualidade de vida do indivíduo.

**Conclusões:**

O emprego do escore de cálcio mostrou-se mais custo-efetivo que a estratégia convencional tanto em custo como em QALY, na maioria dos cenários estudados.

## Introdução

Graças as novas formas de classificar o risco de eventos cardiovasculares na prevenção primária, que são recomendadas pelas diretrizes das principais sociedades de cardiologia do mundo, observa-se aumento significativo na população elegível para o uso de estatinas.^1,2^ Como exemplo dessas mudanças, a diretriz de controle de dislipidemias de 2018 e de prevenção cardiovascular de 2019 da American Heart Association (AHA) e do American College of Cardiology (ACC) sugere o uso de um escore de risco cardiovascular (*Pooled Cohort Equations* — ASCVD) para estimar o risco de eventos cardiovasculares relacionados à aterosclerose em um período de dez anos.^3,4^ Esse escore classifica o indivíduo, de acordo com variáveis modificáveis e não modificáveis, em alto risco (>20% de eventos em dez anos); moderado risco (7,5–20% de eventos em dez anos); *borderline* (5–7,5% de eventos em dez anos) e baixo risco (<5% de eventos em dez anos).^3,4^

Entretanto, é possível notar que essa classificação une uma população de risco cardiovascular heterogênea, já que uma parcela de indivíduos candidatos ao uso de estatina não apresenta sintomas ou sinais de doença aterosclerótica manifesta. Por consequência, muitos dos indivíduos elegíveis à terapia farmacológica poderiam se beneficiar marginalmente dessa terapêutica em longo prazo, uma vez que o benefício acumulado do tratamento é diretamente proporcional ao risco de base.^2,5^

Nesse cenário, o escore de cálcio coronariano (ECC), realizado por meio da tomografia computadorizada para quantificar a carga aterosclerótica dos indivíduos, pode ser útil para reclassificar o paciente intermediário para baixo ou alto risco de eventos, evitando ou, eventualmente, até intensificando a necessidade da terapia hipolipemiante nessa população.^3,4,6,7^

Dessa forma, é relevante a avaliação da efetividade e da custo-efetividade dessa ferramenta em comparação a outros mecanismos de estratificação de risco da população, com o objetivo de orientar a prática clínica, bem como direcionar estrategicamente os esforços e recursos da saúde.

Diversos estudos de custo-efetividade compararam o emprego do ECC à terapia guiada por escores de risco ou outros métodos de classificação.^5,8-11^ Entre eles, Nasir et al.^5^ estudaram a custo-efetividade do emprego do ECC e compararam-na com a estratificação guiada apenas pelo escore de risco de eventos cardiovasculares. Essa análise utilizou os dados e os custos previstos nos Estados Unidos e teve como base os dados populacionais do *Multi-Ethnic Study of Atherosclerosis* (MESA), uma coorte composta de 6.814 participantes de diversos centros de estudos do país.^12^ Neste artigo, usamos como referência o estudo citado anteriormente, com a mesma base populacional citada, adaptando os custos para a realidade brasileira, a fim de verificar a reprodutibilidade do método no Brasil.

## Métodos

**Figura 1:**
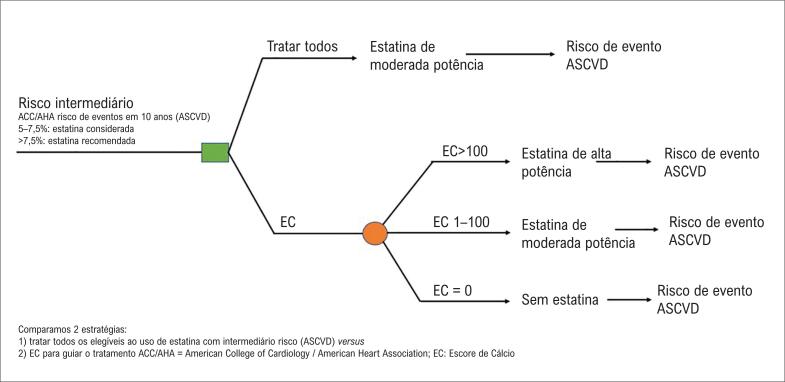
Estratégias para a estratificação do risco em pacientes de risco intermediário.

Nesta análise, a metodologia é replicada do artigo publicado por Nasir et al., sendo realizada por um modelo de microssimulação (TreeAge Pro *version* 2016 — Williamstown, Massachusetts). O modelo simula as consequências clínicas e econômicas da doença cardiovascular aterosclerótica, no contexto da prevenção primária em paciente de moderado risco cardiovascular. As estratégias comparadas nesta análise são (Figura 1) explicitadas a seguir.

Estratégia 1 (convencional): pacientes não realizaram ECC e foram submetidos à terapia farmacológica com estatina de moderada potência.

Estratégia 2 (ECC): pacientes realizaram um ECC, e o tratamento foi guiado pelo resultado. Indivíduos com ECC 1–100 foram submetidos ao tratamento com estatina de moderada intensidade.

Com valor de ECC superior a 100, era iniciado tratamento com estatina de alta potência. Contudo, se ECC:0, não era iniciado tratamento medicamentoso.

A intensidade do tratamento com estatinas, classificada em baixa, moderada e alta potência, segue os critérios contidos na diretriz da AHA e da Sociedade Brasileira de Cardiologia (SBC).^4,13^ As demais medicações de uso contínuo, caso indicadas, não sofreram modificações após a reclassificação de risco.

A análise comparativa do estudo de custo-efetividade é baseada nos anos de vida ajustados por qualidade (*quality-adjusted life years* — QALY) como medida de benefício. A QALY é uma medida de resultado de saúde, que combina em um índice numérico a quantidade (mortalidade) e a qualidade (morbidade) de vida da população, sendo útil para comparar e analisar o resultado comparativo entre as estratégias 1 e 2.

A população desta análise, como citado, é baseada no estudo MESA, e a característica populacional e a distribuição do escore de cálcio de acordo com o risco cardiovascular, baseado nos escores da ACC/AHA, estão demonstradas na Tabela 1 e na Tabela 2.

**Tabela 1:** Característica e distribuição do escore de cálcio coronariano da população do estudo *Multi-Ethnic Study of Atherosclerosis* nas categorias de risco cardiovascular

	Estatina recomendada (n=2.377)	Estatina considerada (n=538)
Idade (anos)	64,7 +-3	58,4 +-6,5
Masculino	1.434 (60)	299 (51)
**Etnia**		
Branco	795 (33)	220 (37)
Negro	791 (33)	180 (31)
Hispânico	534 (23)	124 (21)
Asiáticos	527 (11)	65 (11)
Diabetes	472 (20)	0 (0)
Hipertensão	1.439 (61)	193 (33)
**Fumante**		
Nunca	1.023 (43)	280 (47)
Ex-fumante	918 (39)	211 (36)
Atual	436 (18)	98 (17)
História familiar de DAC	948 (43)	237 (43)
IMC (kg/m2)	28,7 +-5,3	38,5+-5,4
Colesterol total (mg/dl)	201,5 +-34,8	199,8 +-30,6
LDL-C (mg/dl)	126,4 +-31,2	124,6 +-26,4
HDL-C (mg/dl)	48,5 +-13,8	49,9 +- 13,9
Triglicerídeos	132,8 +- 67	126,4 +- 64,4

Valores descritos em média +- DP ou n(%). IMC: índice de massa corporal; DAC: doença arterial coronária; LDL-C: colesterol da lipoproteína de baixa densidade; HDL-c: colesterol da lipoproteína de alta densidade.

**Tabela 2:** Distribuição dos valores de escore de cálcio coronariano de acordo com diretrizes do American College of Cardiology e da American Heart Association

Estatina recomendada	2.377
EC 0	878 (33,0)
EC 1–100	714 (24,1)
EC >100	685 (23,1)
**Estatina considerada**	**598**
EC 0	338 (11,4)
ECC 1–100	184 (6,2)
EC>100	67 (2,3)
Total	2.966 (100)

Valores são n ou n (%). EC: escore de cálcio; ECC: escore de cálcio coronariano.

Nesta investigação, os pacientes percorreram o modelo até apresentar um evento cardiovascular ou morte por outras causas, e o número de anos de uso de estatina ou evento cardiovascular foi pesquisado para cada paciente. O horizonte temporal foi atualizado com ciclos de um ano. Todos os custos e resultados foram descontados em 3% ao ano.

Como limitação do nosso estudo, ressaltamos que a análise dos pressupostos não foi realizada, já que, neste caso, os resultados são extensões de estudos realizados previamente.

### Custos

Como citado anteriormente, houve adaptação dos custos para a realidade brasileira. Os valores estão demonstrados na Tabela 3, em reais (R$) e, em razão da alta variabilidade, estão representados na tabela em três escalas: mediano, mínimo e máximo. Dessa forma, nossa análise foi conduzida com ampla margem de suposições.

**Tabela 3:** Tabela de custos brasileiros

Variável (TreeAge Pro *version* 2016 – Williamstown, Massachusetts)	Mediana (R$)	Mín (R$)	Máx (R$)	Fonte
Exame de ECC	418	300	713	1
Estatina (dose moderada), gasto anual	276,96	210,96	804	2
Estatina (dose intensiva), gasto anual	435,84	324,6	725,64	3
Estatina (todas as doses, mediana), gasto anual	356,4	267,78	764,82	4
Infarto fatal	9.816,8	7.853,44	11.780,16	5
Infarto não fatal, primeiro ano	28.048	22.438,4	33.657,6	6
Infarto não fatal, demais anos	4.207,2	3.365,76	5.048,64	7
Parada cardíaca ressuscitada	42.072	33.657,6	50.486,4	8
AVC fatal	12.761,84	10.209,472	15.314,208	9
AVC não fatal, primeiro ano	56.096	44.876,8	67.315,2	10
AVC não fatal, demais anos	5.890,08	4.712,064	7.068,096	11
Complicações leves de estatinas	650	520	780	12
Complicações importantes de estatinas	19.500	15.600	23.400	13
Seguimento de investigação por achados incidentais não cardíacos (repetição de imagem)	240	200	340	14
Seguimento de consulta médica e testes laboratoriais (revisão ECC, painel lípídico, painel hepático)	80	65	130	15

ECC: escore de cálcio coronariano; AVC: acidente vascular cerebral.

É importante ressaltar que o custo do ECC é acrescentado ao modelo apenas uma vez, já que a repetição do exame não é frequente. Tem-se na literatura que o *warrant time*, ou seja, o tempo de garantia do ECC para indivíduos com ECC=0, é relativamente longo além de individualizado, levando-se em consideração diversos elementos, como idade, sexo e presença de fatores de risco como o diabetes. Sendo assim, em caso de EC zero, a indicação de sua repetição é variável e pode estar indicada em intervalos de três a sete anos.^14^

O restante dos parâmetros clínicos, incluindo análises de sensibilidade de múltiplos parâmetros realizados de forma probabilística, foi utilizado conforme descrito na publicação anterior.

## Resultados

Ao compararmos a custo-efetividade do emprego do ECC na estratificação cardiovascular da prevenção primária do indivíduo de moderado risco cardiovascular entre as estratégias 1 e 2, observamos que, ao se considerar o custo mediano de todas as estatinas e do EC, houve redução estatisticamente significativa de R$ 672,00 nos custos acumulados em favor do grupo que realizou o ECC (Tabela 4 — caso base). Da mesma maneira, ao se reduzir o custo da estatina à mediana das estatinas de moderada intensidade, permanece a diferença de custo acumulado no valor de R$ 423,00, também favorável à realização do ECC. Em outra análise, observamos que além do benefício financeiro se observa maior sobrevida ajustada para QALY, o que corrobora a custo-efetividade do método em relação à estratégia convencional guiada pelas diretrizes.

**Tabela 4:** Parâmetros iniciais para o modelo de microssimulação comparando estratégias de terapia com estatina para indivíduos com risco intermediário de evento cardiovascular

	Custo ECC	Custo Estatina	Custo total por diretrizes	ECC — Custo total	Dif Custo	Diretrizes — QALY	ECC — QALY	QALY Diferença	Conclusão
Caso base	R$ 418,00	R$ 356,00	R$ 6.160,00 (95% IC: 5,587–6,757)	R$ 5.488,00 (95% IC: 4,900–6,113)	-R$ 672,00	11.849 (95% IC: 10.834–12.829)	11.859 (95% IC: 10.859–12.838)	0,01	ECC domina
Caso com estatinas em dose moderada	R$ 418,00	R$ 276,00	R$ 5.492,00 (95% IC: 2,035–10,651)	R$ 5.069,00 (95% IC: 743–10,730)	-R$ 423,00	11.849 (95% IC: 10.834–12.829)	11.859 (95% IC: 10.859–12.838)	0,01	ECC domina

ECC: escore de cálcio coronariano; IC: intervalo de confiança.

**Figura 2:**
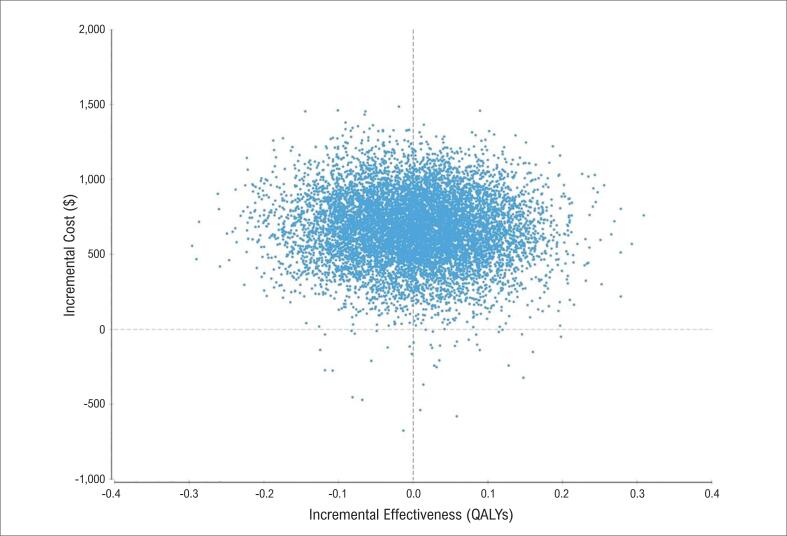
Simulações de Monte Carlo, com 10 mil análises multivariáveis.

Considerando-se as múltiplas variáveis apresentadas, também foram realizadas 10 mil simulações de Monte Carlo para ilustrar a análise de sensibilidade probabilística dos múltiplos parâmetros incluídos no modelo (Figura 2). O gráfico em questão analisa a utilização da estratégia convencional, ou seja, a não utilização do EC na estratificação, por meio de um ganho incremental de QALY no eixo X e os custos incrementais ($ — em moeda local de reais) no eixo Y. Cada ponto no gráfico representa um cruzamento das 10 mil simulações possíveis. Logo, é possível inferir que, utilizando a estratégia convencional de estratificação desses indivíduos, mais de 95% das combinações estão associadas a um ganho incremental de custo sem um ganho incremental de QALY, ou seja, são favoráveis à utilização do EC. Assim, há benefício financeiro ao se comparar a estratégia convencional à estratégia que utiliza o ECC. Entretanto, ao se analisar o QALY, nota-se maior dispersão das simulações, o que não demonstra diferença clara entre as estratégias utilizadas na análise de sensibilidade, apesar de discreta tendência de favorecimento ao grupo que foi submetido ao ECC.

## Discussão

Temos, portanto, com base nos resultados desta análise ajustados para os custos brasileiros, dados que se demonstram favoráveis à utilização da estratégia 2, ou seja, o uso do ECC no auxílio à estratificação cardiovascular e na indicação de estatina, com melhor custo-efetividade em comparação à estratégia 1 (conservadora).

Ao compararmos a custo-efetividade do emprego do ECC como ferramenta de auxílio na estratificação do risco em pacientes em prevenção primária e risco moderado de eventos cardiovasculares, compreendemos os seus reais benefícios e sua aplicabilidade na prática clínica. Os fatores que respaldam esta análise são: 1) a redução do custo incremental de cada estratégia; e 2) o aumento no QALY, que corresponde, em número, ao benefício incorporado à qualidade de vida do indivíduo.

O resultado encontrado nesta pesquisa está de acordo com a literatura, mesmo após o ajuste dos custos para a realidade brasileira. Dessa maneira, estratificar indivíduos de risco moderado para eventos cardiovasculares com o ECC e, com base no resultado obtido, decidir entre o uso ou não da estatina comprova ser vantajoso se comparado à estratégia conservadora.

Dessa forma, restringe-se o número de indivíduos elegíveis ao tratamento medicamentoso e, por consequência, a possibilidade de efeitos adversos relacionados à droga. Ao mesmo tempo, inicia-se o tratamento do indivíduo com real benefício de seu uso e, portanto, podem-se prevenir eventos cardiovasculares associados à aterosclerose. Fica, portanto, evidente a custo-efetividade da estratégia que inclui a utilização do ECC na estratificação desses indivíduos, como ferramenta de extrema importância quando implementada em larga escala.
