# Supplementary material for: Custo-Efetividade do Emprego do Escore de Cálcio Coronariano na Orientação para a Decisão Terapêutica na Prevenção Primária, na População Brasileira
Source: Arq Bras Cardiol. 2022 Jun 6;118(6):1126–31. [Article in Portuguese] doi: 10.36660/abc.20210347 (PMC9345162; doi:10.36660/abc.20210347)
Supplement: Translation

# Translation: Cost-Effectiveness of Using the Coronary Artery Calcium Score in Guiding Therapeutic Decisions in Primary Prevention in the Brazilian Population

**Authors:** Raul Serra Valério, Giuliano Generoso, Juliano Lara Fernandes, Khurram Nasir, Jonathan C. Hong, Marcio Sommer Bittencourt

**Affiliations:** 1 São Paulo SP Brazil Diagnósticos da América SA, São Paulo, SP – Brazil; 2 São Paulo SP Brazil Hospital Sírio-Libanês - Centro de Cardiologia, São Paulo, SP – Brazil; 3 Campinas SP Brazil Radiologia Clínica de Campinas, Campinas, SP – Brazil; 4 Campinas SP Brazil Instituto de Ensino e Pesquisa Jose Michel Kalaf, Campinas, SP – Brazil; 5 Houston Texas USA Houston Methodist Debakey Heart & Vascular Center - Center for Outcomes Research, Houston, Texas – USA; 6 Texas USA Texas Heart Institute - Division of Cardiovascular Surgery, Texas – USA; 7 São Paulo SP Brazil Universidade de São Paulo - Hospital Universitário de São Paulo, São Paulo, SP – Brazil

**Keywords:** Cost-Benefit Analysis, Primary Prevention, Cardiac Imaging Techniques, Coronary Artery Calcium

## Abstract

**Background:**

The use of the coronary artery calcium score to aid cardiovascular risk stratification may be a more cost-effective tool than the conventional strategy.

**Objectives:**

Evaluation of the cost-effectiveness of the use of the calcium score in therapeutic guidance for primary cardiovascular prevention.

**Methods:**

A microsimulation model to assess the clinical and economic consequences of atherosclerotic cardiovascular disease, comparing the prevention strategy using the calcium score and the conventional strategy.

**Results:**

The results obtained demonstrated a better cost-effectiveness of the therapeutic strategy guided by the calcium score, by reducing incremental costs and increasing quality-adjusted life years (QALY), which corresponds, in number, to improving the quality of life of the individual.

**Conclusions:**

The use of the coronary artery calcium score proved to be more cost-effective than the conventional strategy, both in terms of cost and QALY, in most of the scenarios studied.

Thanks to new ways of classifying the risk of cardiovascular events in primary prevention, which are recommended by the guidelines of the main cardiology societies in the world, there is a significant increase in the population eligible for the use of statins.^1,2^ As an example of these changes, the 2018 dyslipidemia management and 2019 cardiovascular prevention guidelines of the American Heart Association (AHA) and American College of Cardiology (ACC) suggest the use of a cardiovascular risk score (Pooled Cohort Equations, ASCVD) to estimate the risk of cardiovascular events related to atherosclerosis over a period of ten years.^3,4^ This score classifies the individual, according to modifiable and non-modifiable variables, as being at high risk (>20% of events in ten years); moderate risk (7.5–20% of events in ten years); borderline (5–7.5% of events in ten years) and low risk (<5% of events in ten years).^3,4^

However, it is possible to note that this classification unites a heterogeneous cardiovascular risk population, since a portion of individuals who are candidates for statin use do not show symptoms or signs of overt atherosclerotic disease. Consequently, many individuals eligible for pharmacological therapy could marginally benefit from this therapy in the long term, since the accumulated benefit of the treatment is directly proportional to the baseline risk.^2,5^

In this scenario, the coronary artery calcium score (CAC), performed by means of computed tomography to quantify the atherosclerotic burden of individuals, may be useful to reclassify the intermediate patient to low or high risk of events, avoiding or eventually even intensifying the need for lipid-lowering therapy in this population.^3,4,6,7^

Thus, it is important to evaluate the therapeutic effectiveness and cost-effectiveness of this tool in comparison to other mechanisms of risk stratification of the population, with the objective of guiding clinical practice, as well as strategically directing health efforts and resources.

Several cost-effectiveness studies have compared the use of CAC to therapy guided by risk scores or other classification methods.^5,8–11^ Among them, Nasir et al. studied the cost-effectiveness of using CAC and compared it with stratification guided only by the risk score for cardiovascular events. This analysis used data and expected costs in the United States and was based on population data from the Multi-Ethnic Study of Atherosclerosis (MESA), a cohort composed of 6,814 participants from different study centers in the country.^12^

Here, we used the aforementioned study as a reference, with the same population base mentioned, adapting the costs to the Brazilian reality, to determine the reproducibility of the method in Brazil.

## Methods

In this analysis, the methods were replicated from the article published by Nasir et al., using a microsimulation model (TreeAge Pro version 2016 — Williamstown, MA, USA). The model simulates the clinical and economic consequences on the basis of atherosclerotic cardiovascular disease, in the context of primary prevention in patients with moderate cardiovascular risk. The strategies compared in this analysis are (Figure 1) explained below.

**Figure 1:** Strategies for risk stratification in intermediate-risk patients.

Strategy 1 (conventional): patients did not undergo CAC and were submitted to pharmacological therapy with moderate-intensity statin.

Strategy 2 (CAC: The CAC was determined in patients, and treatment was guided by the outcome. Subjects with CAC 1–100 underwent moderate-intensity statin treatment.

With a CAC value greater than 100, treatment with high-intensity statin was started. However, with CAC 0, drug treatment was not started.

The intensity of treatment with statins, classified as low-, moderate- and high-intensity, follows the criteria contained in the guidelines of the AHA and the Brazilian Society of Cardiology (SBC).^4,13^ The other medications for continuous use, if indicated, were not modified after the risk reclassification.

The comparative analysis of the cost-effectiveness study was based on quality-adjusted life years (QALY) as a measure of benefit. QALY is a health outcome measure, which combines the population's quantity (mortality) and quality (morbidity) of life in a numerical index, being useful to compare and analyze the comparative result between strategies 1 and 2.

The population of this analysis, as mentioned, is based on the MESA study, and the population characteristics and distribution of the calcium score according to cardiovascular risk, based on the ACC/AHA scores, are shown in Tables 1 and 2.

**Table 1:** Characteristics and distribution of coronary artery calcium score in the Multi-Ethnic Study of Atherosclerosis population based on cardiovascular risk categories

	Statin recommended (n=2,377)	Statin considered (n=538)
Age (years)	64.7 ± 3	58.4 ± 6.5
Male	1,434 (60)	299 (51)
**Ethnicity**		
While	795 (33)	220 (37)
Black	791 (33)	180 (31)
Hispanic	534 (23)	124 (21)
Asian	527 (11)	65 (11)
Diabetes	472 (20)	0 (0)
Hypertension	1.439 (61)	193 (33)
**Smoker**		
Never	1,023 (43)	280 (47)
Former	918 (39)	211 (36)
Current	436 (18)	98 (17)
Family history of CAD	948 (43)	237 (43)
BMI (kg/m2)	28.7± 5.3	38.5 ± 5.4
Total cholesterol (mg/dl)	201.5 ± 34.8	199.8 ± 30.6
LDL-C (mg/dl)	126.4 ± 31.2	124.6 ± 26.4
HDL-C (mg/dl)	48.5 ± 13.8	49.9 ± 13.9
Triglycerides	132.8 ± 67	126.4 ± 64.4

Values given as mean ± SD or n(%). BMI: body mass index; CAD: coronary artery disease; LDL-C: low-density lipoprotein cholesterol; HDL-c: high-density lipoprotein cholesterol.

**Table 2:** Distribution of coronary artery calcium scores according to American College of Cardiology and American Heart Association guidelines

Statin recommended	2,377
CAC 0	878 (33.0)
CAC 1–100	714 (24/1)
CAC >100	685 (23.1)
**Statin considered**	**598**
CAC 0	338 (11.4)
CAC 1–100	184 (6.2)
CAC >100	67 (2.3)
Total	2,966 (100)

Values are n or n (%). CAC: coronary artery calcium score.

In this investigation, patients were run through the model until they had a cardiovascular event or death from other causes, and the number of years of statin use or cardiovascular event was searched for each patient. The time horizon was updated with one-year cycles. All costs and results were discounted at 3% per year.

As a limitation of our study, we emphasize that the analysis of the assumptions was not performed, since in this case, the results are extensions of studies carried out previously.

### Costs

As previously mentioned, the costs were adapted to the Brazilian reality. The values are shown in Table 3, in reais (R$) and, due to the high variability, they are represented in the table in three scales: median, minimum and maximum. Thus, our analysis was conducted with a wide range of assumptions.

**Table 3:** Brazilian costs

Variable (TreeAge Pro version 2016 – Williamstown, MA, USA)	Median (R$)	Min (R$)	Max (R$)	Source
CAC examination	418	300	713	1
Statin (moderate-intensity), annual cost	276.96	210.96	804	2
Statin (high-intensity), annual cost	435.84	324.60	725.64	3
Statin (all intensities, median), annual cost	356.40	267.78	764.82	4
Fatal infarction	9,816.80	7,853.44	11,780.16	5
Nonfatal myocardial infarction, first year	28,048	22,438.40	33,657.60	6
Nonfatal infarction, other years	4,207.20	3,365.76	5,048.64	7
Cardiac arrest resuscitated	42,072	33,657.60	50,486.40	8
Fatal CVA	12,761.84	10,209.47	15,314.20	9
Nonfatal CVA, first year	56,096	44,876.80	67,315.20	10
Nonfatal CVA, other years	5,890.08	4,712.06	7,068.09	11
Mild complications of statins	650	520	780	12
Major complications of statins	19,500	15,600	23,400	13
Follow-up investigation for non-cardiac findings (repeat imaging)	240	200	340	14
Clinical follow-up and laboratory tests (CAC review, lipid panel, liver panel)	80	65	130	15

CAC: coronary artery calcium score.

It is important to note that the cost of CAC was added to the model only once, as the test is not repeated frequently. In the literature, the warrant time, that is, CAC guarantee time for individuals with CAC=0, is relatively long in addition to being individualized, taking into account several aspects such as age, sex and the presence of risk factors, including diabetes. Therefore, in case of a zero calcium score, the indication of its repetition is variable and may be indicated at intervals of three to seven years.^14^

The rest of the clinical parameters, including probabilistic multiparameter sensitivity analyses, were used as described in the previous publication.

## Results

When comparing the cost-effectiveness of using CAC in the cardiovascular stratification of primary prevention of individuals with moderate cardiovascular risk between strategies 1 and 2, we observed that when considering the median cost of all statins and the CAC, there was a statistically significant reduction of R$ 672.00 in accumulated costs in favor of the group in which CAC was determined (Table 4 – base case). In the same way, when the cost of the statin was reduced to the median of moderate-intensity statins, the accumulated cost difference of R$ 423.00 remained, also favorable to the performance of the CAC. In another analysis, we observed that in addition to the financial benefit, there was a greater QALY survival, which confirmed the cost-effectiveness of the method in relation to the conventional strategy based on the guidelines.

**Table 4:** Parameters for the microsimulation model that compared strategies for statin therapy in individuals at intermediate risk for an ASCVD event

	CAC	Statin cost	Total cost per guidelines	CAC — Total cost	Cost difference	Guidelines — QALY	CAC — QALY	QALY Difference	Conclusion
Base case	R$ 418.00	R$ 356.00	R$ 6.160,00 (95%CI: 5,587–6,757)	R$ 5.488,00 (95%CI: 4,900–6,113)	-R$ 672	11,849 (95%CI: 10,834–12,829)	11,859 (95%CI: 10,859–12,838)	0.01	CAC dominates
Case with moderate-intensity statins	R$ 418.00	R$ 276.00	R$ 5.492,00 (95%CI: 2,035–10,651)	R$ 5.069,00 (95%CI: 743–10,730)	-R$ 423	11,849 (95%CI: 10,834–12,829)	11,859 (95%CI: 10,859–12,838)	0.01	CAC dominates

CAC: coronary artery calcium score; CI: confidence interval.

Considering the multiple variables presented, 10,000 Monte Carlo simulations were also performed to illustrate the probabilistic sensitivity analysis of the multiple parameters included in the model (Figure 2). The graph in question analyzed the use of the conventional strategy, that is, the non-use of CAC in stratification, through an incremental gain of QALY on the X axis and the incremental cost ($ — in local currency of reais) on the Y axis. Each point on the graph represents a cross between the 10,000 possible simulations. Therefore, it is possible to infer that using the conventional strategy of stratification in these individuals, more than 95% of the combinations were associated with an incremental gain in cost without an incremental gain in QALY; that is, they were favorable to the use of CAC. Thus, there was a financial benefit when comparing the conventional strategy to the strategy that used CAC. However, when analyzing QALY, there was a greater dispersion of the simulations, which did not show a clear difference between the strategies used in the sensitivity analysis, despite a slight tendency to favor the group that involved CAC.

**Figure 2:** Monte Carlo simulations with 10,000 multivariate analyses.

## Discussion

Therefore, based on the results of this analysis, adjusted for Brazilian costs, we have data that are favorable to the use of strategy 2, that is, the use of CAC to support cardiovascular stratification and statin indication, with better cost-effectiveness. compared to strategy 1 (conservative).

When comparing the cost-effectiveness of using the CAC as a tool to aid in risk stratification in patients undergoing primary prevention and moderate risk of cardiovascular events, we understand its real benefits and its applicability in clinical practice. The factors that support this analysis are: 1) the reduction of the incremental cost of each strategy; and 2) the increase in QALY, which corresponds, in number, to the benefit incorporated into the individual's quality of life.

The results obtained in this study are in agreement with the literature, even after adjusting the costs to the Brazilian reality. Thus, stratifying individuals at moderate risk for cardiovascular events with CAC and on the basis of the results obtained, deciding whether or not to use a statin proves to be advantageous compared to the conservative strategy.

Thus, the number of individuals eligible for drug treatment is limited and consequently the possibility of adverse drug-related effects. At the same time, treatment of the individual is initiated with real benefit from its use, and therefore, cardiovascular events associated with atherosclerosis can be prevented. Therefore, the cost-effectiveness of the strategy that includes the use of CAC in the stratification of these individuals is evident, as an extremely important tool when implemented on a large scale.
